# Yang-deficiency constitution drives poor outcomes in clear cell renal cell carcinoma by modulating the tumour immune microenvironment

**DOI:** 10.3389/fimmu.2025.1673579

**Published:** 2025-11-20

**Authors:** Boon Seng Kho, Zongyuan Zhou, Rui Liu, Yihang Sui, Yingnan Zhang, Jiaqi Yao, Huanhuan Lu, Guowei Zhou, Bo Zhang, Yinyin Wang

**Affiliations:** 1 Department of Traditional Chinese Medicines (TCMs) Pharmaceuticals, School of Traditional Chinese Pharmacy, China Pharmaceutical University, Nanjing, China; 2 Sichuan Industrial Institute of Antibiotics, School of Pharmacy, Chengdu University, Chengdu, China; 3 Department of General Surgery, Affiliated Hospital of Nanjing University of Chinese Medicine, Nanjing, China

**Keywords:** clear cell real cell carcinoma (ccRCC), pre-diagnosis biomarkers, immuneinfiltration, tumor immune microenvironment, therapeutic intervention, Yang-deficiencyconstitution (YDC)

## Abstract

**Background:**

Clear cell renal cell carcinoma (ccRCC) is the most common subtype of kidney cancer, often diagnosed at advanced stages due to a lack of reliable early biomarkers. Recent studies suggest that the traditional Chinese medicine (TCM) body constitution, particularly the Yang-Deficiency Constitution (YDC), may influence tumour development by altering the immune microenvironment. However, the mechanistic connection between YDC and ccRCC prognosis remains largely unexplored.

**Objective:**

This study aims to elucidate the impact of YDC on the immune landscape and clinical outcomes of ccRCC and to identify novel prognostic biomarkers and potential herbal therapeutic agents guided by YDC characteristics.

**Methods:**

We integrated bulk transcriptomic data from 12 YDC-classified individuals and 530 ccRCC patients, alongside single-cell RNA-seq profiles from one PBMC and two ccRCC tumour samples. Through differential expression analysis, WGCNA, and machine learning-based survival modelling, we identified YDC-related biomarkers and assessed their immunological relevance using ESTIMATE, CIBERSORT, and CellChat. A gene expression-based scoring framework (GSVA) was developed to systematically prioritize 622 herbal ingredient perturbations for their potential survival benefits. Key ingredients were further validated through molecular docking and experimental assays.

**Results:**

Patients with YDC-associated ccRCC exhibited poorer survival. Nine intersecting genes were screened and used to construct a prognostic model, whereby seven key biomarkers—MXD3, PLCB2, CCDC88B, DEF6, IFNG, TBC1D10C, and PLEKHN1—were significantly influenced the prognosis of renal cancer. These genes were found to modulate immune cell populations, particularly CD8^+^ T cells, Tregs, and M1 macrophages, with IFNG serving as a central regulatory hub. Baicalein was identified and validated as a promising therapeutic agent targeting IFNG.

**Conclusion:**

This study highlights the crucial role of YDC in shaping the immune microenvironment and influencing survival in ccRCC. By integrating constitution-based stratification, immune profiling, and herbal medicine screening, we offer a unique framework for biomarker discovery and propose baicalein as a potential YDC-targeted adjuvant therapy.

## Introduction

Clear cell renal cell carcinoma (ccRCC) is the most common and aggressive subtype of kidney cancer, accounting for approximately 70–90% of all renal malignancies (1). Despite progress in diagnostic imaging and molecular testing, ccRCC is frequently diagnosed at advanced stages due to its pronounced molecular heterogeneity and lack of reliable early biomarkers. According to the theory of oncology, biomarkers play essential roles in early detection, risk stratification, prognosis, and therapeutic decision-making (2, 3). For example, HER2 serves as a key biomarker in breast cancer (4), while EGFR mutations guide the development of targeted therapies in non-small cell lung cancer (NSCLC) (5). However, the identification of robust and clinically actionable biomarkers in ccRCC remains challenging, primarily due to limited preclinical validation and the complex biological landscape of the disease (6).

Beyond genetic mutations, the tumour immune microenvironment (TIME) has emerged as a crucial determinant of ccRCC progression and therapeutic response. Immune cell infiltration, cytokine signaling, and stromal interactions collectively shape tumour behaviour by promoting angiogenesis, immune suppression, and metastasis (7). The advent of immune checkpoint inhibitors (ICIs) has significantly improved outcomes in ccRCC by restoring antitumor immunity; however, response rates remain variable, and the mechanisms underlying immune evasion are incompletely understood (8). This variability underscores the need to integrate host-specific factors into immune-based prognostic models.

One such host factor is body constitution; a concept rooted in traditional Chinese medicine (TCM) that categorizes individuals into distinct physiological types with differential susceptibility to disease. Among the nine classical TCM constitutions, the Yang-Deficiency Constitution (YDC) has garnered attention for its association with immune dysfunction and metabolic impairment (9–12). Clinically, individuals with YDC typically exhibit cold intolerance, coldness in the hands, feet, stomach, and waist, a preference for warm food and drinks, and an increased susceptibility to cold exposure; whereas secondary manifestations often include watery stool, whitish skin, nocturia, a pale and tender tongue, and a tendency toward obesity (**Table 1**) (13). Furthermore, the kidney is central to energy metabolism, fluid regulation, and immune balance—functions that closely parallel endocrine and immunological mechanisms recognized in modern medicine (14, 15). Specifically, YDC is characterized by reduced mitochondrial activity, impaired glucose and lipid metabolism, and weakened adaptive immune responses (16), suggesting a potential role in shaping the tumour immune microenvironment and affecting cancer progression (17).

**Table 1:** Diagnosis standard for yang-deficient and balanced constitution (21)

Type of Constitution	Yang-deficient constitution	Balanced constitution
Main features	Cold intolerance Chilly in the extremities and lower back Favor warmth, including hot beverages Vulnerable to cold	Vigorous Without Yang-deficient related symptoms
Secondary features	Loose stool Propensity to gain weight Frequent urination Fair or pale complexion Soft and pale tongue	Good appetite Good sleep Healthy body posture

Emerging evidence supports the relevance of constitutional types in cancer biology. For instance, patients with Yang-deficiency or Phlegm-Dampness constitutions report higher levels of cancer-associated fatigue and systemic inflammation (18). Constitution-guided herbal interventions, such as Tao Hong Si Wu Tang, have demonstrated efficacy in modulating epithelial–mesenchymal transition, angiogenesis, and immune responses in preclinical cancer models (19). Furthermore, constitution types have been linked to disease phenotypes in non-oncologic conditions, such as mild cognitive impairment (20), further supporting their value for patient stratification and precision treatment.

**Figure 1:**
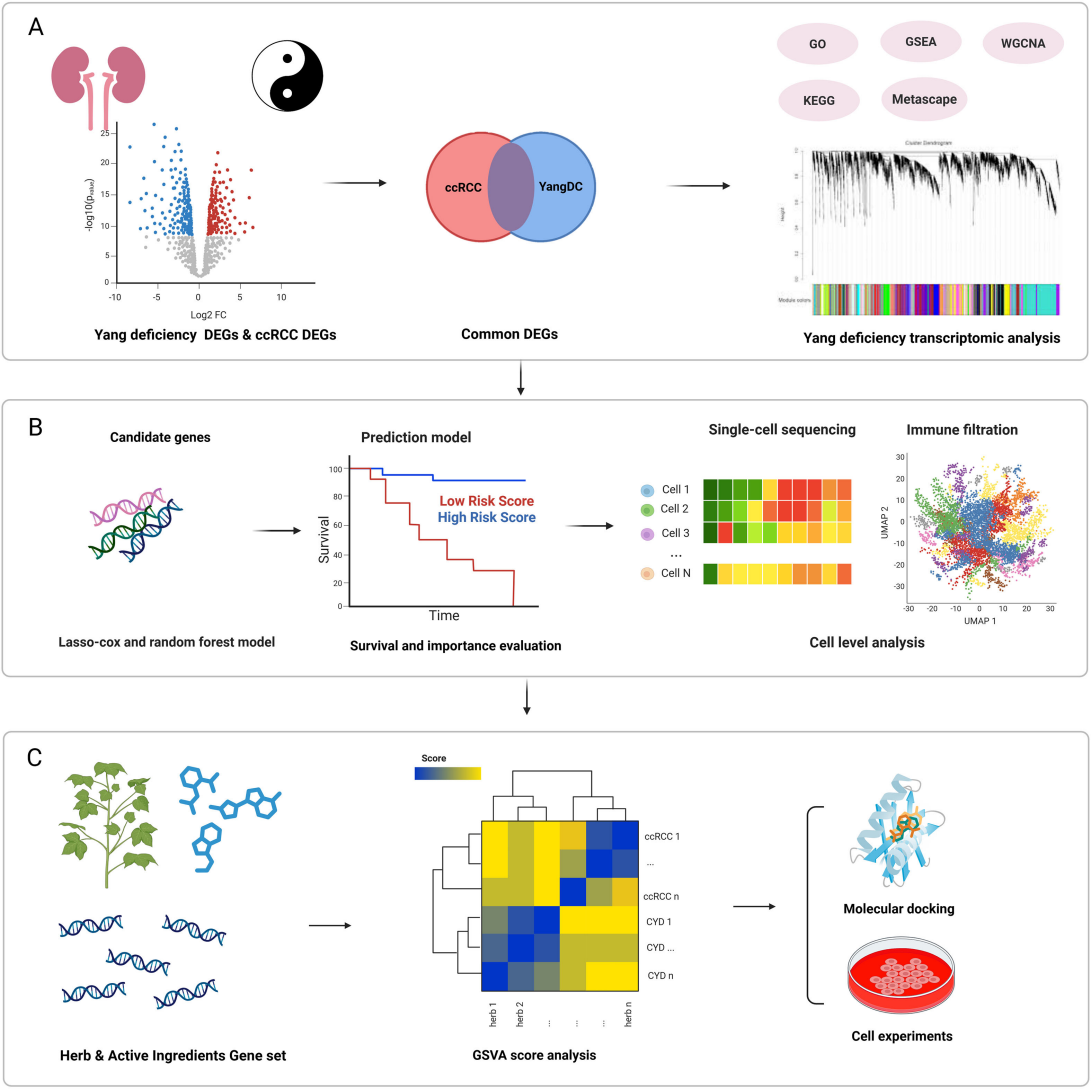
Overview of the study design. **(A)** Functional analysis of ccRCC and YDC. **(B)** Identification of potential prognostic biomarkers and construction of a predictive survival model followed by immune microenvironment characterization using CIBERSORT and CellChat. **(C)** Screening and experimental validation of herbal compounds with potential to prolong ccRCC survival using GSVA scoring, molecular docking, and *in vitro* assays.

Despite increasing interest in constitution theory, the mechanistic connection between YDC and the TIME in ccRCC remains largely unexplored. Given the immunogenic nature of ccRCC, YDC may influence tumour evolution and patient outcomes through immune modulation. However, the underlying molecular networks and therapeutic implications of this relationship are not yet well defined. In this study, we systematically investigated the role of YDC in shaping the immune microenvironment and survival outcomes in ccRCC. As shown in **Figure 1**, we integrated transcriptomic and single-cell RNA-seq data to identify YDC-associated gene signatures (YDGs) and applied machine learning approaches to evaluate their prognostic relevance. Functional characterization of these genes was conducted using immune deconvolution tools (CIBERSORT, ESTIMATE) and cell–cell communication analysis (CellChat), revealing their regulatory roles in immune cell infiltration and signaling. To translate these findings into potential therapeutic strategies, we developed a Gene Set Variation Analysis (GSVA)-based scoring system to prioritize 622 herbal ingredients with predicted benefits on YDC-related signatures. Top candidates were validated via molecular docking simulations and *in vitro* assays.

Collectively, our study highlights the clinical importance of YDC in ccRCC by linking constitution-specific immune dysregulation to poor prognosis. We also propose a novel constitution-guided approach for biomarker discovery and herbal compound screening, offering alternative therapeutic strategies for precision oncology.

## Materials and methods

### Dataset acquisition

Bulk RNA-seq profiles of individuals with YDC and typical controls were obtained from the Gene Expression Omnibus (GEO) (GSE87474, n=20) (21). The ccRCC transcriptomic dataset (n = 534) was accessed via the UCSC Xena platform. Single-cell RNA-seq (scRNA-seq) data were collected from peripheral blood mononuclear cells (PBMCs) of a healthy donor (GSE115189) and two ccRCC tumour samples (GSE152938) (22). Differentially expressed genes (DEGs) related to 622 herbal ingredients were retrieved from the HERB (23) and ITCM (24) databases.

### Differential gene expression and functional enrichment analysis in Yang deficiency samples

DEGs in YDC samples were identified using the *limma* package (25) in R, applying a threshold of |log_2_FC| > 2.5 and *P<* 0.05 due to relatively small cohort distributions and to minimize false positives for the subsequent pathway analyses. Functional enrichment analyses were conducted to reveal the biological relevance of these DEGs. Gene Ontology (GO), Kyoto Encyclopaedia of Genes and Genomes (KEGG), and Gene Set Enrichment Analysis (GSEA) were employed using the clusterProfiler package to identify enriched biological pathways.

### Weighted gene co-expression network analysis in Yang deficiency samples

Weighted Gene Co-expression Network Analysis (WGCNA) was performed using the *wgcna*package in R to identify YDC-associated gene modules (26). The workflow included: (1) hierarchical clustering of samples to detect outliers, (2) determination of soft-thresholding power and adjacency matrix construction, (3) conversion into a topological overlap matrix (TOM), (4) module identification via dynamic tree-cutting (minimum module size = 30), and (5) correlation analysis between modules and the YDC phenotype to identify hub genes for downstream analysis.

### Identification of shared signature genes between YDC and ccRCC

DEGs from the ccRCC and YDC datasets were intersected to identify shared biomarkers. Gene–gene correlation networks were constructed using *igraph* and *ggraph* (27), retaining edges with a Spearman correlation coefficient greater than 0.3. Functional modules were identified using the Louvain community detection algorithm (28) to highlight gene clusters with co-expression patterns. These DEGs were used to quantify the degree of YDC traits in ccRCC samples, thereby providing a semi-quantitative approach to define the YDC cohort. The DEGs associated with YDC were compiled into gene sets for gene set variation analysis (GSVA). Followed by calculating the resulting enrichment scores, which were then used to quantify the transcriptional activity of YDC-related molecular patterns in each ccRCC patient. Patients were subsequently stratified into high- and low-score groups based on the median GSVA score, and survival analyses were performed to assess the association between YDC-related transcriptional signatures and clinical outcomes.

### Survival analysis using LASSO-cox regression

The overlapping genes between YDC and ccRCC were subjected to prognostic evaluation using LASSO-Cox regression (29) via the *glmnet* package (30). Cross-validation determined the optimal regularization parameter (λ). A predictive risk score for each patient was calculated:

(1) $$Risk\;Score_{i}=\sum_{j=1}^{p}\beta_{j\;}*\;x_{ij}$$

Where $\beta_{j}$ is the LASSO-derived coefficient for the gene j and $x_{ij}$ is its expression in sample i. Patients were stratified into high- and low-risk groups for Kaplan–Meier survival analysis using the *ggsurvplot* package.

### Random forest-based survival analysis

A random survival forest model was built using the *randomForestSRC* package (31), with overall survival and status as outcomes and gene expression profiles as input. The model was selected due to its high predictive performance among multiple models for breast cancer survival, along with an accuracy of 96% and an area under the curve (AUC) of 0.93 (32). Furthermore, the model required minimal data preprocessing, was resilient to outliers, and could effectively identify important predictors that inform personalized clinical decision-making, which fulfilled the main idea of the topic (33). In addition, the RSF model provided more individualized survival curves and captured non-constant hazard dynamics over time after comparison with Cox and Support Vector Machine (SVM) models, which is crucial for personalized treatment planning (34). Feature importance was assessed, and the individual prognostic value of each gene was further evaluated using univariate Cox regression (35), receiver operating characteristic (ROC) curves (AUC), and Kaplan–Meier plots.

### Construction of a prognostic prediction model for clinical survival probability at different time intervals

A prognostic nomogram was developed to predict 1-, 2-, and 3-year survival by integrating expression levels of selected biomarkers into a multivariate Cox model. Calibration plots were used to assess the predictive accuracy. Decision curve analysis (DCA) (36) evaluated clinical net benefit across probability thresholds.

### Immune microenvironment analysis of ccRCC samples on cell type proportion

The ESTIMATE algorithm (37) was applied to calculate immune and stromal scores in ccRCC samples. CIBERSORT (38) was used to estimate the relative abundance of 22 immune cell types. Spearman correlation analysis was conducted to examine relationships between prognostic biomarkers and immune cell infiltration. To validate immune associations, immune phenotypic scores (IPS) from The Cancer Immunome Atlas (39) were analysed, stratifying samples by CTLA-4/PD-1 responsiveness.

### Gene expression distribution and bioactivity enrichment of biomarker genes on SC-Seq of ccRCC samples

Two single-cell datasets derived from PBMCs and ccRCC samples were utilized to investigate cellular activity and gene expression dynamics. Two scRNA-seq datasets were analysed using *Seurat* (40) for normalization, dimensionality reduction (principal component analysis, PCA), and clustering (uniform manifold approximation and projection, UMAP). Low-quality cells with<200 or >5, 000 detected genes or mitochondrial content >10% were removed. Cell-type annotation was performed using *SingleR* (41) or PBMCs and CellMarker (42) for ccRCC tumours. Cell types were annotated by calculating the average expression of known biomarker genes in each cluster and matching them to established immune and tumour marker sets from the CellMarker databases. Clusters showing the highest marker concordance were assigned cell identities. Only markers with log_2_FC > 0.25, *P* < 0.05, and min.pct > 0.1 were retained. Activity of YDC-related gene signatures in PBMCs was assessed via *AddModuleScore* (43) while the Area Under the Curve cell (*AUCell*) (44) quantified signature activity in ccRCC single cells based on AUC scores.

### Cellchat analysis to explore the dynamics of cellular interaction of biomarker genes on SC-Seq of ccRCC samples

To investigate intercellular communication patterns among immune and stromal cells, we employed the *CellChat* from the R package (45) using single-cell transcriptomic data. We constructed a CellChat object using the normalized expression matrix and cell-type annotations and inferred cell–cell communication networks based on a curated ligand–receptor interaction database. The interactions between cell types were quantified and visualized using circle plots. To explore immune-related signaling, we focused on the prognostic gene-related signaling pathway, where communication probabilities and ligand–receptor interactions were evaluated and visualized through chord plots and dot plots. Furthermore, the role of each cell type in the pathway, as sender, receiver, mediator, or influencer, was assessed. Lastly, a heatmap summarizing outgoing and incoming signal strengths was generated to highlight the dominant signaling populations. All analyses and visualizations were performed using the standard CellChat workflow and its built-in plotting functions.

### Screening for therapeutic herbal ingredients using GSVA

Herbal ingredient–associated gene sets were retrieved from HERB and ITCM. Firstly, the DEGs obtained from the HERB database were analysed using the *limma* package, while genes from the ITCM database were directly retrieved. To minimize false positives, only differentially expressed genes (DEGs) with *P* < 0.05 were used. The resulting genes were curated and compiled into herb-specific gene sets for enrichment analysis. GSVA was used to evaluate enrichment scores of these gene sets within each sample. For sample j, the enrichment score $s_{kj}$ for herb j was calculated as:

(2) $$s_{kj}=max_{r}\left(P_{in}\left(r\right)-P_{out}\left(r\right)\right)\;$$

where $\left(P_{in}\left(r\right)\;and\;P_{out}\left(r\right)\right)$ represent empirical cumulative distribution functions (ECDFs) for gene expression ranks inside and outside gene set $G_{k}$, respectively. This score summarizes the pathway activity of herbal ingredients across samples. Top-ranking ingredients were considered for downstream therapeutic validation.

### Molecular docking simulation to confirm the binding between the potential ingredient and the targeted biomarkers

To validate the therapeutic potential of the identified compound, molecular docking simulations were performed. The 3D structural data of the herbal-derived compound baicalein were obtained from the PubChem chemical substance database (46). At the same time, the crystal structure of the key biomarker protein IFNG was retrieved from the RCSB Protein Data Bank (47). Protein–ligand docking simulations were conducted using PyMOL and AutoDock Tools (48), and the binding affinity between baicalein and IFNG was estimated to assess their potential interaction.

### Cell experimental validation

#### Cell lines and culture conditions

The human renal carcinoma cell line 786-O (purchased from Nanjing Runyan Biotechnology Co., Ltd.) was routinely cultured under sterile conditions at 37°C in a humidified atmosphere containing 5% CO_2_. Cells were grown in RPMI-1640 medium supplemented with 10% heat-inactivated fetal bovine serum (FBS) and 1% penicillin-streptomycin. Cell lines were passaged upon reaching 80-90% confluence using 0.25% trypsin-EDTA solution and subculture at appropriate seeding densities. Culture medium was refreshed every 2–3 days, and cells were routinely monitored for morphology and mycoplasma contamination.

#### Cell viability assays

786-O cells were plated in 96-well microplates (5 × 10³ cells/well) containing 100 μL complete growth medium and incubated for 12–16 hr at 37°C under 5% CO_2_ to achieve cellular adhesion. After adherence confirmation, cells were treated with gradient concentrations of baicalein or vehicle control (0.1% DMSO) for specified durations. At the treatment endpoint, 10 μL of CCK-8 solution (MedChemExpress, Shanghai, China) was added to each well without replacing the medium. Plates were subjected to orbital agitation (30 sec, 100 rpm) to ensure reagent dispersion and then further incubated at 37°C and 5% CO_2_ for precisely 2 hours. Optical density was subsequently quantified at dual wavelengths (450 nm test; 650 nm reference) using a Varioskan LUX microplate reader (Thermo Scientific) to correct for nonspecific absorption.

#### Cell apoptosis and cycle distribution analysis

Following baicalein incubation, 786-O cells were harvested and resuspended to generate a single-cell suspension. Cells were pelleted via centrifugation (1000 × g, 3 min). After supernatant aspiration, the cell pellet was washed once with 1 mL ice-cold phosphate-buffered saline (PBS), transferred to a 1.5 mL microcentrifuge tube, and centrifuged again to form a pellet. After supernatant removal, the cells were fixed by dropwise addition of 1 mL ice-cold 70% ethanol under gentle agitation and then incubated at 4°C for a minimum of 30 minutes. Fixed cells were recovered by centrifugation (1000 × g, 3 min). Following careful aspiration of the ethanol, the cell pellet was resuspended in 0.5 mL of propidium iodide (PI) Staining Solution (Beyotime Biotechnology, Shanghai, China) and incubated at 37 °C in the dark for 30 min. Stained samples were maintained at 4 °C in the dark until analysis. Apoptosis assessment was performed by quantifying Relative Fluorescence Units (RFU) using 488 nm excitation with Varioskan LUX (Thermo). Cell cycle distribution was analyzed using the BriCyte^®^ E6 flow cytometer (Mindray), and subsequent quantification of cell cycle phases was performed using ModFit LT 5.0 software.

### Statistical analysis

All analyses were performed using R version 4.4.2. Gene correlations were assessed using either Spearman or Pearson correlation coefficients, as appropriate. Survival analyses were conducted using Kaplan–Meier estimation and Cox proportional hazards regression. Unless otherwise specified, a p-value< 0.05 was considered statistically significant.

## Results

### Biological characteristics of Yang-deficiency constitution

**Figure 2:**
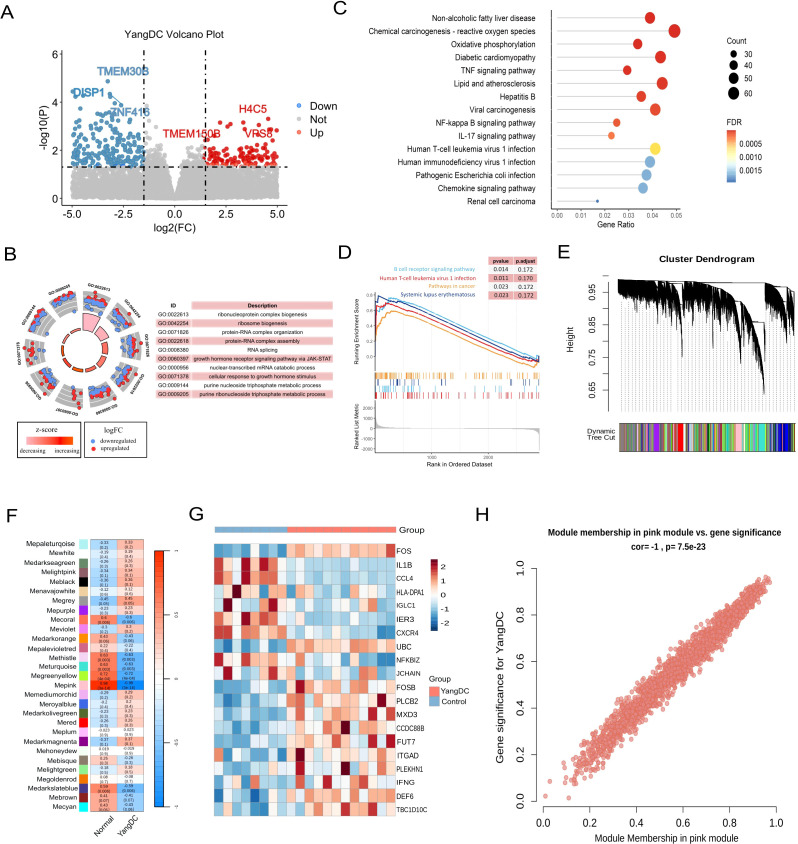
Transcriptomic profiling of YDC. **(A)** Volcano plot showing differentially expressed genes (DEGs) in YDC versus healthy controls (|log_2_FC| > 2.5, *P* < 0.05). **(B)** Gene Ontology (GO) enrichment analysis of DEGs. **(C)** KEGG pathway enrichment of DEGs. **(D)** GSEA analysis revealing pathway enrichment in YDC. **(E)** Gene co-expression modules derived from WGCNA. **(F)** Correlation between modules and YDC phenotype to identify the key module. **(G)** Heatmap of gene expression from the YDC-associated module. **(H)** Scatter plot of module membership (MM) and gene significance (GS) for hub gene identification.

To elucidate the biological characteristics of the YDC, we analysed RNA-seq data from 12 individuals with YDC and eight healthy controls using the GSE87474 dataset. Differential expression analysis revealed 29, 555 differentially expressed genes (DEGs) in YDC samples compared to healthy individuals (**Figure 2A**, **Supplementary Table 1**). GO enrichment analysis indicated that these DEGs are predominantly involved in ribonucleoprotein complex biogenesis, protein-RNA complex organization, and mitochondrial inner membrane organization (**Figure 2B**). KEGG pathway analysis further demonstrated that YDC is associated with altered biological functions in pathways such as amyotrophic lateral sclerosis, lipid metabolism, atherosclerosis, Salmonella infection, and the TNF signaling pathway (**Figure 2C**). Additionally, GSEA highlighted significant enrichment in immune- and cancer-related pathways, including the B cell receptor signaling pathway, cancer pathways, and systemic lupus erythematosus (**Figure 2D**). These results collectively suggest a potential link between YDC and cancer development and progression.

To further identify gene modules closely associated with the YDC phenotype, we employed WGCNA, which clustered genes into 29 modules based on topological overlap (**Figure 2E**). Among these, the MEpink module exhibited the strongest correlation with the YDC trait (r = 0.98, *P<* 0.01), and thus was chosen for further interpretation. (**Figure 2F**). Moreover, genes in this module show distinct expression across samples (**Figure 2G**). Notably, chemokines such as *CXCR4* and *CCL20*, both located within the pink module, are known to promote carcinogenesis, angiogenesis, and the survival of cancer cells. Elevated CCL20 expression has also been associated with poor prognosis in hepatocellular carcinoma (HCC) patients following curative resection (49, 50). These findings support the involvement of pink module genes in cancer-related pathological processes. This association was further corroborated by a strong positive correlation between module membership (MM) and both the YDC trait and gene significance (GS), with a Pearson correlation coefficient of R = 1.0 and p = 7.5e^-23^ (**Figure 2H**).

In summary, our integrative RNA-seq analysis of YDC and healthy individuals identified significant dysregulation in immune and cancer-associated pathways. We also identified a key gene module comprising 2, 058 genes—referred to hereafter as the YDC signature genes—that may play critical roles in the onset and progression of cancer in individuals with a Yang-deficiency Constitution.

### Association between YDC and ccRCC and their pre-diagnostic biomarker genes

**Figure 3:**
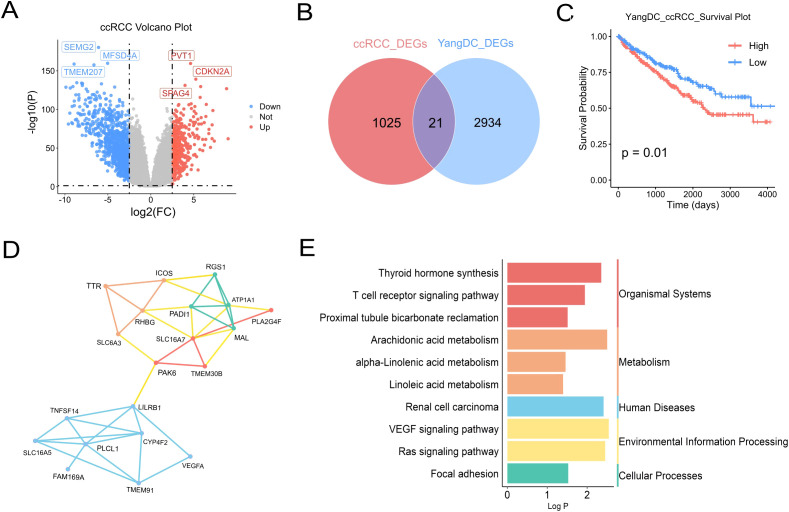
Association of YDC with ccRCC progression and survival. **(A)** Volcano plot showing DEGs in ccRCC versus healthy tissues (|log_2_FC| > 2.5, *P* < 0.05). **(B)** Venn diagram showing overlapping genes between ccRCC and YDC-related DEGs. **(C)** Kaplan–Meier survival analysis of ccRCC patients stratified by YDC activity. **(D)** Gene co-expression network of common DEGs associated with YDC and ccRCC. **(E)** KEGG enrichment of overlapping genes, highlighting ccRCC-relevant pathways.

Having established that YDC significantly influences the survival of patients with ccRCC, we further explored the functional association between YDC and ccRCC. A total of 1, 047 DEGs were identified when comparing ccRCC samples to healthy controls (**Figure 3A**, **Supplementary Table 2**). Among these, 21 genes overlapped with those associated with YDC (**Figure 3B**), highlighting a potential mechanistic link.

Survival analysis revealed that ccRCC patients with YDC exhibited significantly poorer prognosis (**Figure 3C**), further supporting a functional association between YDC and disease progression. Functional enrichment analysis using the Metascape database showed that these 21 overlapping genes were significantly involved in bile salt, organic acid, metal ion, and amine compound transport, as well as in monocarboxylic acid transport, positive regulation of cell adhesion, circulatory system processes, and the Ras signalling pathway (**Supplementary Figure 1**).

Gene co-expression network analysis of the 21 YDC-associated genes revealed two major gene clusters (**Figure 3D**). The first cluster, comprising TNFSF14, LILRB1, PLCL1, CYP4F2, and VEGFA, was primarily associated with immune regulation and inflammatory signaling, suggesting an immunological component to YDC in ccRCC. The second cluster, including SLC16A7, ATP1A1, RGS1, MAL, and PADI1, was enriched in transmembrane transport and metabolic processes, indicating a potential link between YDC and metabolic reprogramming. Notably, PAK6 and TMEM30B appeared in both clusters and may serve as key regulatory hubs connecting immune and metabolic pathways. Pathway enrichment analysis based on the KEGG database further demonstrated that these genes were significantly enriched in pathways related to organismal systems, metabolism, human diseases, environmental information processing, and cellular processes. Specifically, the renal cell carcinoma pathway, T cell receptor signaling pathway, linoleic acid metabolism, and VEGF signaling pathway—each previously implicated in ccRCC—were prominently enriched (**Figure 3E**).

**Figure 4:**
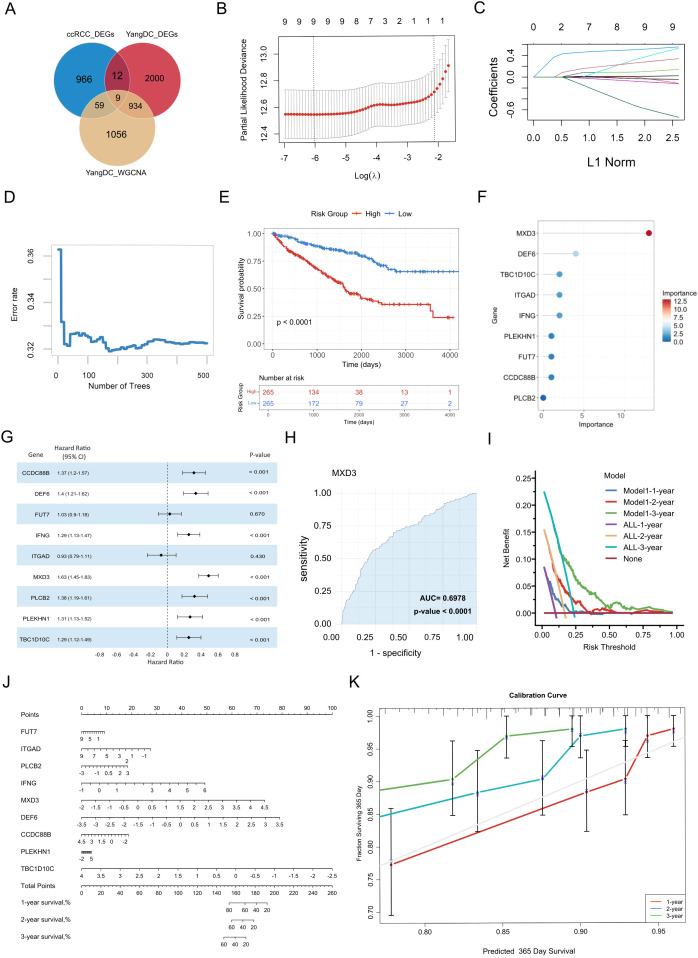
Identification and validation of YDC-related prognostic biomarkers in ccRCC. **(A)** Overlapping genes from WGCNA and DEGs were used for biomarker discovery. **(B)** Feature selection via LASSO regression with cross-validation. **(C)** LASSO coefficient profiles showing how each gene’s regression coefficient changes with increasing L1 penalty. **(D)** Error rate trend with increasing tree number in LASSO. **(E)** Kaplan–Meier curves for high- vs. low-expression groups. **(F)** Feature importance scores of selected biomarkers. **(G)** Hazard ratio estimates of biomarker genes. **(H)** Diagnostic performance of MXD3 via ROC analysis. **(I)** Decision curve analysis evaluating model performance across time points. **(J)** Nomogram for predicting individual survival probability. **(K)** Calibration curves assessing nomogram accuracy for 3-year survival.

By integrating the signature genes associated with YDC and ccRCC, we identified nine overlapping candidate genes: MXD3, DEF6, IFNG, TBC1D10C, CCDC88B, ITGAD, PLEKHN1, PLCB2, and FUT7 (**Figure 4A**).

Collectively, these findings highlight the potential biological relevance of YDC in the pathogenesis and progression of ccRCC, with the nine overlapping genes identified as candidate signature genes associated with YDC-mediated regulation of ccRCC.

### Evaluation of pre-diagnostic biomarker genes and predictive model for ccRCC survival risk

To evaluate the prognostic potential of these genes, we employed LASSO regression analysis to determine the optimal regularization parameter (λ) and identify genes with significant survival relevance. Cross-validation results indicated that the minimum partial likelihood deviance occurred around log(λ) ≈ −6 (**Figure 4B**), confirming all nine genes as non-zero contributors to the predictive model. The coefficient trajectories demonstrated that several genes maintained non-zero coefficients despite increasing regularization (**Figure 4C**), indicating their stability and prognostic robustness.

Subsequently, a random forest model was constructed to quantify gene importance and assess predictive performance. The model’s prediction error decreased rapidly within the first 100 decision trees and stabilized after approximately 200 trees (**Figure 4D**). Finally, the 500 trees were selected as the final model parameter to ensure a stable estimation. Using this approach, the nine genes were scored by their contribution to model prediction. Then, Kaplan–Meier survival analysis further demonstrated that patients in the high-risk group, as the risk scores had been evaluated by Equation 1 - classified based on the risk scores derived from these genes—had significantly worse survival than those in the low-risk group (log-rank test, *P<* 0.001) (**Figure 4E**). Additionally, MXD3 was identified as the most critical prognostic gene, with the highest variable importance score (20) and a hazard ratio (HR) of 1.63 (*P* < 0.001) (**Figures 4F, G**). DEF6 and IFNG also ranked highly, suggesting strong associations with survival outcomes.

ROC curve analysis confirmed the diagnostic efficacy of individual biomarkers, with MXD3 achieving the highest area under the curve (AUC = 0.70, *P* < 0.001). Most genes exhibited moderate but statistically significant discriminatory power (**Figure 4H**, **Supplementary Figure 2**). All nine genes were significantly associated with overall survival (HR > 1, *P<* 0.001), further validating their prognostic potential.

To enhance the clinical utility of the identified biomarkers, we constructed a gene-based nomogram model for predicting 3-year survival risk in ccRCC (**Figure 4J**). The nomogram assigns a weighted score to each gene, with the total score corresponding to estimated survival probability. Calibration plots demonstrated strong concordance between predicted and observed survival rates, especially for 1-year predictions, indicating robust model calibration (**Figure 4K**).

Moreover, DCA demonstrated that the nomogram offers a greater net clinical benefit across a threshold probability range of 0.25 to 0.60 (**Figure 4I**), underscoring the clinical applicability of the seven most predictive genes in long-term survival estimation.

In summary, these findings highlight nine YDC-related genes as promising pre-diagnostic biomarkers for evaluating long-term survival risk in ccRCC patients. Building on this, we developed and validated a prognostic model based on YDC-associated gene expression, providing a robust and clinically applicable tool for survival risk prediction in ccRCC.

### Immune infiltration analyses of prognostic genes in the ccRCC cohort

To investigate the potential immune-related mechanisms through which YDC influences the prognosis of ccRCC, we conducted comprehensive analyses of the immune microenvironment. Given the central role of immune dysregulation in tumour progression, we utilized single-cell transcriptomic data to examine the immune context of prognostic biomarkers.

**Figure 5:**
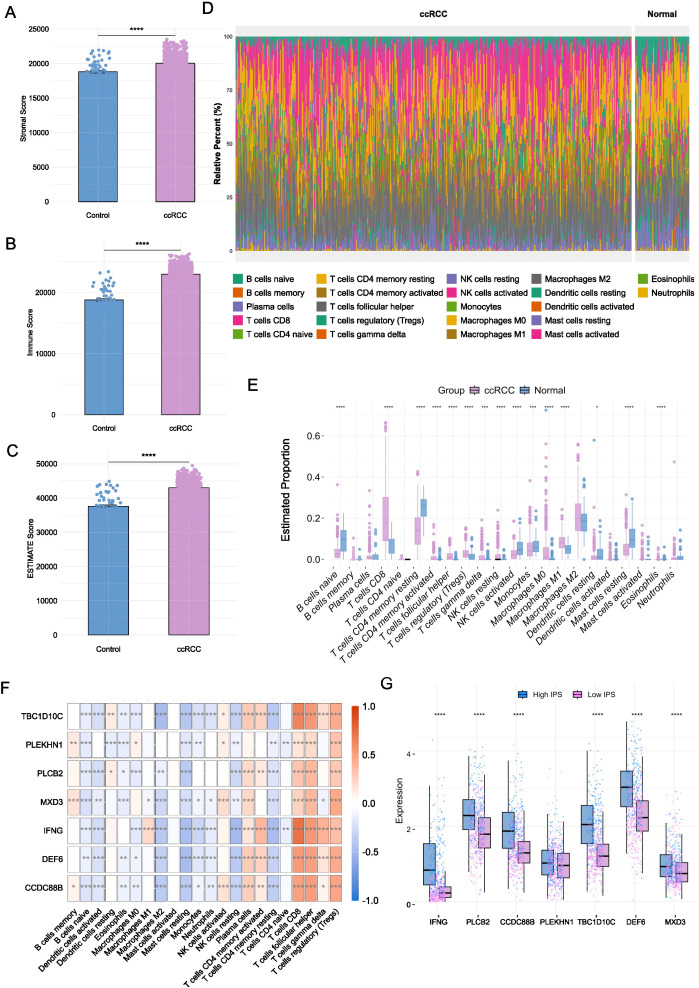
Immune microenvironment characterization to prognostic biomarkers. **(A-C)** Immune, stromal, and ESTIMATE scores in ccRCC versus healthy tissues (*P < 0.05, **P < 0.01, ***P < 0.001, ****P < 0.0001, Mann-Whitney test). **(D)** Distribution of 22 immune cell types across groups. **(E)** Differential abundance of immune cell types between ccRCC and healthy samples (*P < 0.05, **P < 0.01, ***P < 0.001, ****P < 0.0001, n-Whitney test). **(F)** Spearman correlations between biomarker gene expression and immune cell infiltration. **(G)** Comparison on the expression levels of biomarker genes across high and low IPS groups (*P < 0.05, **P < 0.01, ***P < 0.001, ****P < 0.0001, n-Whitney test).

First, we applied the ESTIMATE algorithm to infer the proportions of stromal and immune components in the tumour microenvironment based on gene expression data. The analysis revealed significantly elevated immune, stromal, and composite ESTIMATE scores in ccRCC samples compared to healthy controls (**Figure 5A-C, P** < 0.001), indicating a profoundly altered tumour microenvironment in ccRCC. To further characterize the immune cell composition, we employed the CIBERSORT algorithm to deconvolute the bulk transcriptomic profiles into 22 distinct immune cell types (**Figure 5D**). ccRCC tissues exhibited increased infiltration of immune effector and regulatory cells, including CD8^+^ T cells, regulatory T cells (Tregs), monocytes, and M1 macrophages (**Figure 5E, P** < 0.001). In contrast, healthy samples displayed higher levels of naïve B cells, activated NK cells, and resting dendritic cells.

To explore the relationship between immune infiltration and our identified prognostic genes, we performed Spearman correlation analyses to assess the association between gene expression and immune cell infiltration scores. The YDC-related prognostic genes demonstrated strong positive correlations with key immune subsets (**Figure 5F, P** < 0.001). Similarly, NK cells activated showed a positive correlation with IFNG, TBC1D10C, CCDC88B, and DEF6 (**Figure 5F, P** < 0.001). For example, CD8^+^ T cell and Treg infiltration levels were positively correlated with most biomarkers, while NK cell activation showed significant correlations with IFNG, TBC1D10C, CCDC88B, and DEF6. These findings suggest that the identified genes may serve as modulators of immune cell recruitment and inflammatory signaling in the tumour microenvironment.

To validate these findings, we used IPS from the external TCIA database. Samples were stratified into high-IPS and low-IPS groups, and gene expression was compared across groups (**Supplementary Table 3**). All nine prognostic biomarkers were significantly upregulated in the high-IPS group (**Figure 5G, P** < 0.001), reinforcing their association with enhanced immune responsiveness.

Taken together, our immune infiltration analyses suggest that these YDC-associated biomarkers may influence ccRCC patient survival by modulating the immune microenvironment, particularly through the regulation of CD8^+^ T cells, Tregs, and M1 macrophages.

### Single-cell resolution of biomarker expression and functional enrichment

**Figure 6:**
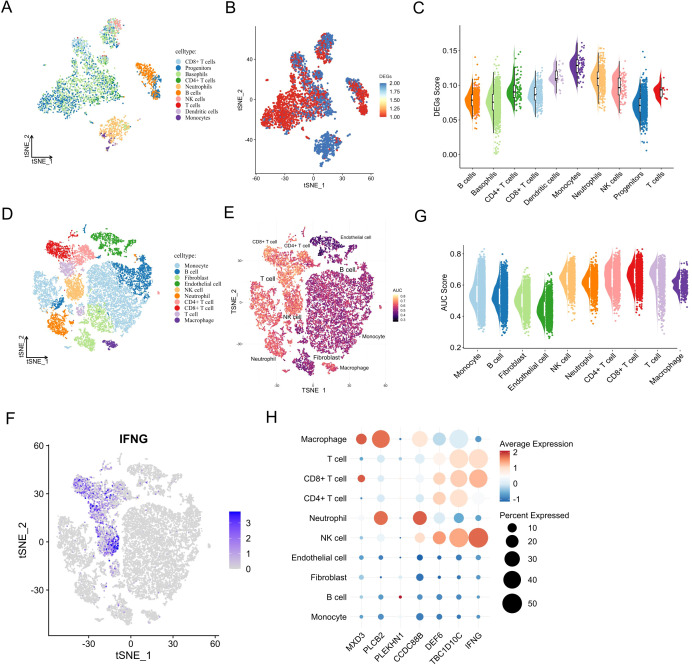
Single-cell expression patterns and biological roles of prognostic biomarkers. **(A)** UMAP of annotated cell types in healthy PBMCs. **(B, C)** Activity score distribution of YDC-related DEGs in single-cell data. **(D)** UMAP of cell clusters in the ccRCC single-cell RNA-seq dataset. **(E)** AUC-based pathway activity scores for YDC–ccRCC gene sets. **(F)** UMAP projection of IFNG expression. **(G)** AUC scores across cell types in the ccRCC single-cell RNA-seq dataset. **(H)** Expression and distribution of prognostic biomarkers by cell type.

To gain a high-resolution view of the immune-related functions of the prognostic biomarkers, we analysed single-cell RNA sequencing (scRNA-seq) data from PBMCs and ccRCC tumour samples. In the PBMC dataset, 3, 372 cells were classified into 10 significant immune populations, including CD8^+^ T cells, CD4^+^ T cells, B cells, NK cells, dendritic cells, monocytes, neutrophils, progenitors, basophils, and general T cell subsets (**Figure 6A**). Using a manually curated gene set based on YDC-related DEGs, we calculated enrichment scores for each cell type using the AddModuleScore function. Monocytes and neutrophils exhibited the highest module scores (**Figures 6B-C**), suggesting their involvement in YDC-associated immune alterations.

Similarly, analysis of 20, 599 cells from ccRCC tumour tissues revealed that YDC-related gene sets were significantly enriched in CD8^+^ T cells, NK cells, T cells, and macrophages (**Figures 6D-E**), with the strongest signals in T cells and NK cells (**Figures 6F-G**). These enrichment points to the critical involvement of these immune cell types in mediating YDC-related effects within the tumour microenvironment.

Examining the distribution of individual prognostic genes across cell types further supported their immune relevance (**Figures 6F-H**, **Supplementary Figure 3**). For instance, TBC1D10C was highly expressed in NK cells, Tregs, and T cells, while DEF6 showed strong expression in NK cells and Tregs. These expression patterns suggest a likely role for these genes in modulating the immune response and inflammation within the tumour niche.

Together, these findings reveal that the seven YDC-related prognostic genes are not only functionally associated with immune infiltration but are also preferentially expressed in key immune cell subsets at the single-cell level. This highlights their potential role in immune remodelling and tumour progression in ccRCC.

### Cellular and molecular interactions of prognostic biomarkers in the tumour microenvironment

To elucidate the cellular and molecular dynamics of biomarker interactions within the ccRCC tumour microenvironment, we conducted CellChat analysis using single-cell RNA sequencing data (GEO152938). Understanding these interactions is essential for uncovering pathogenic mechanisms and identifying novel therapeutic targets.

**Figure 7:**
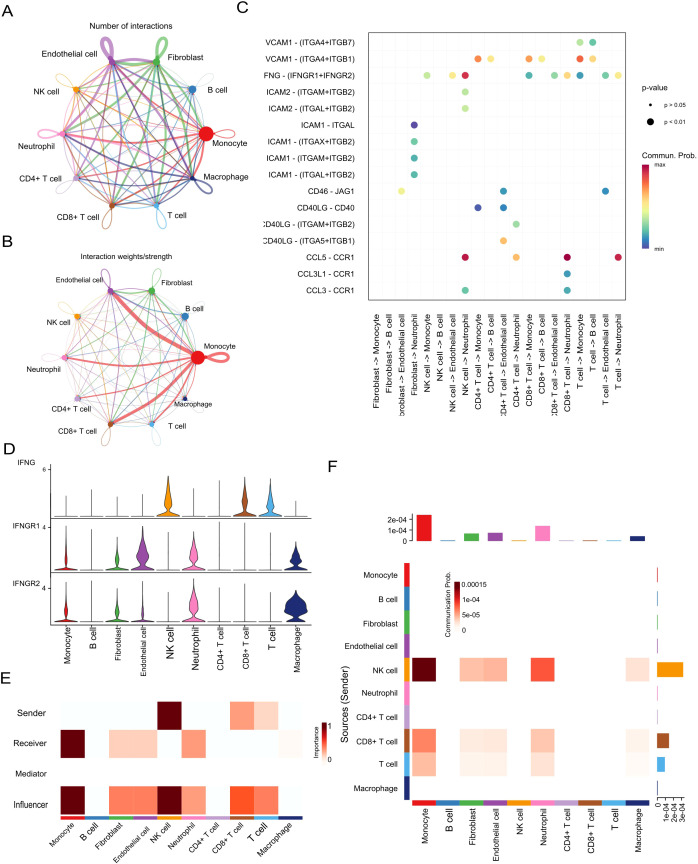
Cell–cell communication and IFNG signalling in the ccRCC tumour microenvironment. **(A, B)** Intercellular communication networks based on interaction number and strength. **(C)** Key ligand–receptor interactions identified across cell populations. **(D)** Expression levels of IFNG, IFNGR1, and IFNGR2 in different cell types. **(E)** Summary of IFN-II pathway interaction scores. **(F)** Outgoing and incoming signalling roles of the IFN-II pathway.

The overall cell–cell communication network revealed extensive crosstalk among immune and stromal cell populations (**Figure 7A**). Among these, monocytes and macrophages emerged as central hubs—monocytes exhibited the highest number of interactions, while macrophages demonstrated the strongest interaction weights (**Figure 7B**), suggesting their dominant regulatory roles in the ccRCC immune milieu.

Notably, IFNG, one of the identified prognostic biomarkers, was found to be the primary ligand initiating interferon-γ (IFN-γ) signaling, particularly mediating interactions between NK cells and CD8^+^ T cells (**Figure 7B**). In this signaling axis, monocytes and macrophages functioned as key receptors, forming a pivotal regulatory loop in innate–adaptive immunity. Quantitative ligand–receptor interaction analysis further confirmed a high communication probability for the IFNG–IFNGR1/2 pair (**Figure 7C**), underscoring the central role of IFNG in modulating immune responses.

Functional role mapping revealed that monocytes were not only dominant signal receivers but also strong influencers, whereas NK cells primarily served as signal senders and influencers (**Figure 7D**). This dynamic suggests a feedback mechanism in which IFNG derived from NK cells activates monocytes, contributing to an inflammatory tumour microenvironment.

Expression analysis of IFNG and its receptors corroborated these findings: IFNG was highly expressed in NK and CD8^+^ T cells, while its receptors (IFNGR1 and IFNGR2) were broadly distributed across monocytes, macrophages, fibroblasts, and endothelial cells (**Figure 7E**). Heatmap analysis of IFN-II signaling strength revealed prominent communication from NK and CD8^+^ T cells toward monocytes and macrophages (**Figure 7F**), reinforcing the centrality of IFNG-mediated signaling in shaping the immune landscape of ccRCC.

Collectively, these findings highlight IFNG as both a key prognostic biomarker and a functional orchestrator of intercellular immune communication within the ccRCC tumour microenvironment, particularly by coordinating crosstalk between innate and adaptive immune components.

### Identification of herbal medicines targeting prognostic biomarkers to improve ccRCC outcomes and experimental validation

Building upon the identification of nine prognostic biomarkers associated with YDC and ccRCC, we aimed to identify herbal medicines capable of modulating the expression of these biomarkers to improve clinical outcomes.

**Figure 8:**
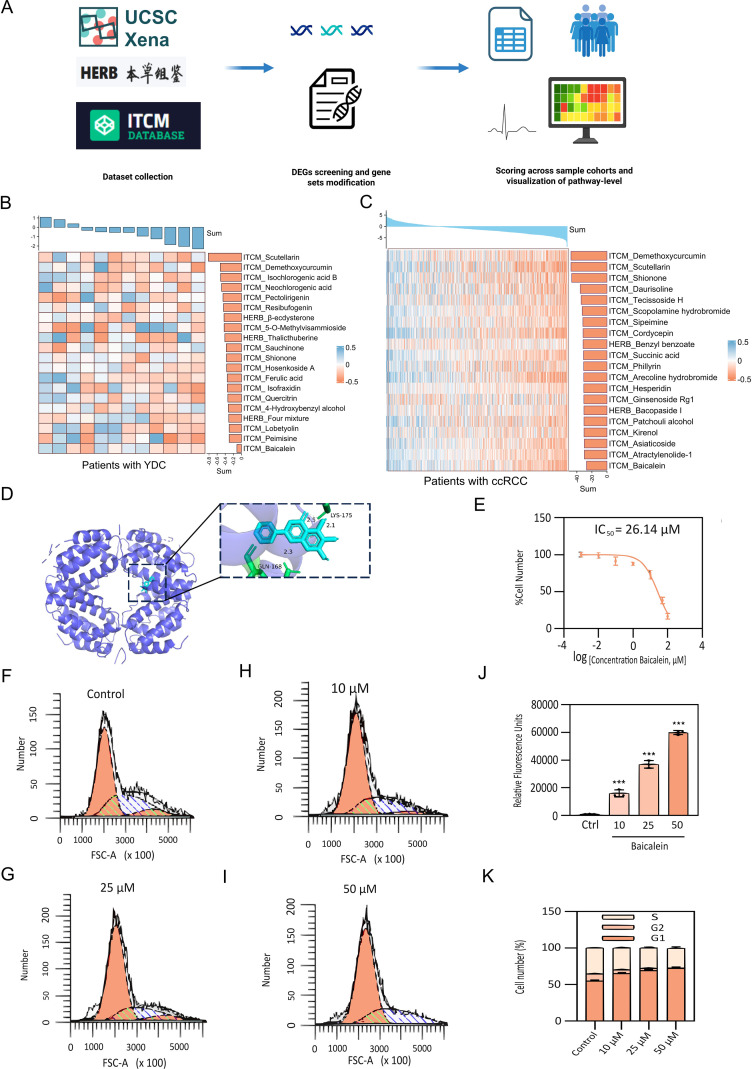
Identification and experimental validation of herbal therapeutics targeting YDC–ccRCC biomarkers. **(A)** Workflow for GSVA-based herbal compound screening. **(B-C)** Regulatory activity of candidate herbal compounds on YDC and ccRCC-related signatures. **(D)** Molecular docking between IFNG and baicalein, showing stable binding. **(E)** Dose-dependent inhibition of 786-O cell proliferation by baicalein (CCK-8 assay). **(F-I)** Cell cycle analysis following 24h baicalein treatment (10, 25, 50 μM). **(J)** Apoptosis induction in 786-O cells after baicalein exposure, assessed via PI staining. **(K)** Quantification of apoptosis and cell cycle arrest. Data shown as mean ± SD (n = 3). ***P< 0.001 vs. control.

A high-throughput screening was performed using perturbation data for 622 herbal medicines and their active compounds, curated from the HERB and ITCM databases. To prioritize candidate compounds, we computed an importance score derived from GSVA, evaluating the enrichment of YDC-related and ccRCC gene signature across herbal ingredients by Equation 2 (**Figure 8A**). Compounds with negative enrichment scores—indicating inverse regulation of disease-associated genes—were considered potential therapeutic candidates. Notably, scutellarin and baicalein emerged as top candidates, demonstrating robust and consistent downregulation of YDC- and ccRCC-associated gene signatures (**Figures 8B, C**).

To assess mechanistic plausibility, molecular docking analyses were conducted to evaluate the binding affinity of these compounds with interferon gamma (IFNG), one of the key hub genes identified in our prognostic model. Scutellarin was predicted to bind within the active site of IFNG stably. Similarly, baicalein formed multiple hydrogen bonds—particularly with GLN-168 and LYS-175—indicating a favourable binding conformation and strong molecular interaction (**Figure 8D** and **Table 2**). These in silico findings suggest that both compounds may modulate IFNG activity, thereby influencing immune-related signaling pathways and survival outcomes in ccRCC.

**Table 2:** Summary of molecular docking results of baicalein and INFG protein.

Run	Binding energy (kcal/mol)	Intermolecular energy (kcal/mol)
1	-5.37	-7.46
2	-5.30	-7.39
3	-5.05	-7.14
4	-4.90	-6.99
5	-4.85	-6.94
6	-4.83	-6.92
7	-4.80	-6.89
8	-4.71	-6.80
9	-4.31	-6.61

To experimentally validate the antitumor potential of baicalein, we conducted *in vitro* assays using human renal carcinoma 786-O cells. A CCK-8 cell viability assay revealed that baicalein significantly inhibited cell proliferation in a dose-dependent manner, with a half-maximal inhibitory concentration (IC_50_) of 26.14 μM (**Figure 8E**). Flow cytometric analysis further demonstrated a marked increase in apoptotic cell populations following baicalein treatment at concentrations of 10, 25, and 50 μM (**Figure 8J**), indicating that baicalein not only suppresses cell proliferation but also promotes apoptosis.

To further elucidate the mechanism of growth inhibition, we analyzed cell cycle distribution. Untreated 786-O cells exhibited a balanced distribution among G1, S, and G2/M phases (**Figure 8F**). Following baicalein treatment (**Figures 8G–I**), a dose-dependent accumulation of cells in the G1 phase was observed, increasing from 55.13% ± 1.21% (control) to 72.40% ± 1.39% at 50 μM (**Figure 8K**). This was accompanied by a corresponding reduction in S and G2/M populations, suggesting that baicalein induces G1 phase arrest, thereby inhibiting DNA synthesis and mitotic progression. These findings demonstrate that baicalein exerts a dual anti-tumor effect in ccRCC cells by inhibiting proliferation through G1 phase arrest and promoting apoptosis.

Collectively, our integrative analysis and experimental validation underscore the therapeutic potential of baicalein as a promising natural compound that targets YDC-related biomarkers for the improved management of ccRCC.

## Discussion

In this study, we systematically explored the prognostic value of YDC in ccRCC, integrating transcriptomic profiling, machine learning algorithms, immune landscape deconvolution, and herbal medicine screening. Our results highlight that YDC is not only a clinically relevant constitutional phenotype but also exerts a profound influence on the tumour immune microenvironment (TIME) and survival outcomes in patients with ccRCC.

We identified a robust gene signature associated with YDC that stratifies patient survival risk and demonstrates strong predictive power across multiple cohorts. Notably, these YDC-related genes (YDGs) exhibit immune cell-type specificity and are functionally enriched in immune modulation, cell adhesion, bile acid transport, and Ras signalling—biological processes that are frequently dysregulated in ccRCC (51). These findings suggest that YDC may drive tumour-promoting phenotypes via immune dysregulation, metabolic reprogramming, and chronic inflammation, offering a constitution-based framework for precision oncology. Complementary pathway enrichment and GSEA analyses revealed that individuals with YDC show elevated activity in chemical carcinogenesis, ROS and lipid metabolism, TNF signalling, and ribosome biogenesis—pathways associated with metabolic abnormalities, oxidative stress, immune suppression, and tumour proliferation (52–57). Moreover, enrichment in B-cell receptor (BCR) signalling and systemic lupus erythematosus (SLE)-related gene sets suggests a heightened inflammatory state and potential autoimmunity risk, both of which are epidemiologically linked to renal cancer (58, 59). YDC-related genes were particularly enriched in pathways regulating bile salt transport, cell adhesion, and Ras signalling—all of which are crucial for maintaining renal homeostasis and are commonly dysregulated in ccRCC (60, 61).

Importantly, the identified YDGs are not only prognostically significant but also mechanistically linked to TIME modulation. Through CIBERSORT and single-cell RNA sequencing analyses, we observed elevated infiltration of CD8^+^ T cells, T regulatory cells (Tregs), NK cells, and M2 macrophages in the YDC subgroup. While CD8^+^ T cells typically confer anti-tumour immunity, their activity is often impaired under nutrient-deprived and immunosuppressive microenvironments - a hallmark of ccRCC (62). Several YDGs—including IFNG, CCDC88B, DEF6, PLCB2, and TBC1D10C—were found to modulate key immune functions, such as antigen presentation, TCR signalling, macrophage activation, and cytokine production (63–67). Furthermore, IFNG emerged as a central immunoregulatory hub, mediating both immune activation (via MHC-I and chemokine induction) and immune exhaustion under chronic stimulation (68). These insights reinforce the hypothesis that YDC reflects a constitution-driven immunometabolism state that shapes tumour progression and responsiveness to immune-based therapies. This positions YDC as not just a prognostic indicator, but a potentially actionable factor in tailoring ccRCC treatment strategies.

Furthermore, our study presents a novel and unique panel of prognostic biomarkers that capture the dynamic interactions between body constitution, the tumour, and its immune microenvironment. Using LASSO and random forest models, we identified nine YDGs with superior stratification ability compared to conventional biomarkers. These genes, such as MXD3, DEF6, and PLEKHN1, are implicated in processes including DNA repair, lipid metabolism, epithelial–mesenchymal transition (EMT), and immune suppression (69–71). Their enrichment in specific immune cell populations (T cells and macrophages) underscores their role in modulating TIME and promoting tumour immune evasion.

In addition to providing a prognostic framework, we also developed an innovative herbal compound screening strategy tailored to YDC biology. By integrating transcriptomic perturbation profiles from over 600 herbal medicines, we prioritized compounds that reverse the YDC-related gene expression signature. Two leading candidates, baicalein and scutellarin, demonstrated consistent suppression of disease-related pathways and strong binding affinity to IFNG, confirmed by molecular docking. Functional validation further demonstrated that baicalein inhibited ccRCC cell proliferation, induced G1-phase cell cycle arrest, and promoted apoptosis, thereby supporting its therapeutic potential in targeting both immune modulation and tumour survival mechanisms (72).

Despite the promising findings, several limitations should be taken into consideration. First, our analyses relied heavily on transcriptomic data from public databases, which may introduce cohort-specific biases and statistical robustness. In order to address this issue, we applied empirical Bayes moderation and module-based co-expression analysis to reduce bias; however, future validation may require balanced cohorts. Second, although YDC was analysed as a dominant constitutional type, body constitution is multifaceted, and future studies should explore its interaction with other TCM syndromes. In addition, the acknowledgement of RNA-seq transcriptomic data was insufficient in capturing protein-level regulation. Consequently, proteomic and immunohistochemical validation of the biomarkers will be essential in future discovery. The uneven cohort distribution and subjective selection of the machine-learning models might contribute to potentially biased conclusions. Therefore, future analytical workflows should incorporate multiple machine learning models and adopt more stringent thresholds to enhance the objectivity and robustness of the results. Baicalein was computationally identified as a potential compound targeting YDC-related genes, as the current study did not include *in vitro* or *in vivo* validation to confirm whether baicalein modulates IFNG expression or signalling pathways. Future mechanistic studies, such as cell-based assays, will be necessary to verify this regulatory mechanism. The pharmacodynamics and synergistic effects of herbal compounds remain underexplored due to incomplete data on ingredient–target interactions. Comprehensive *in vivo* validation and mechanistic investigations are warranted to elucidate the therapeutic value of these compounds fully.

Collectively, our findings provide a preliminary framework for predicting survival risk and exploring constitution-guided therapeutic strategies in ccRCC. While the proposed YDC-based stratification may offer complementary insights to existing precision-oncology approaches, its clinical utility remains to be further validated. Future prospective and experimental studies are warranted to confirm the translational relevance of these findings and to evaluate whether constitution-guided interventions can meaningfully improve patient outcomes.

## Conclusions

In summary, this study reveals the prognostic and immunological relevance of YDC-related traits in ccRCC. By integrating bulk and single-cell transcriptomic analyses, we identified a YDC-associated gene signature with strong predictive value, particularly MXD3, DEF6, PLCB2, TBC1D10C, IFNG, CCDC88B, and PLEKHN1, which are important in immune modulation as a potential mechanism underlying poor prognosis in YDC-phenotypic renal cancer patients. Moreover, baicalein was computationally predicted as a promising herbal compound that may target YDC-related genes to regulate immune responses and inhibit tumour progression. While similar integrative approaches have been explored in related contexts, our work provides an additional perspective linking traditional constitutional theory with immune-oncological mechanisms, suggesting a potential direction for constitution-informed precision medicine in renal cancer, which requires further clinical and experimental evaluation.

## Data Availability

The datasets presented in this study can be found in online repositories. The names of the repository/repositories and accession number(s) can be found in the article/**Supplementary Material**.

## Supplementary material

Supplementary Figure 1 Metascape pathway enrichment of common DEGs between YDC and ccRCC.

Supplementary Figure 2 The survival validation of biomarkers. (A-F) ROC curves for prognostic biomarker validation. (G-M) Survival analyses for individual prognostic genes.

Supplementary Figure 3 UMAP projections showing single-cell expression distributions of key prognostic genes.

Supplementary Table 1 Yang deficiency DEGs.

Supplementary Table 2 ccRCC DEGs.

Supplementary Table 3 ccRCC TCIA clinical data.
