# Supplementary figures and images for: Yang-deficiency constitution drives poor outcomes in clear cell renal cell carcinoma by modulating the tumour immune microenvironment
Source: Front Immunol. 2025 Nov 20;16:1673579. doi: 10.3389/fimmu.2025.1673579 (PMC12675363; doi:10.3389/fimmu.2025.1673579)

A

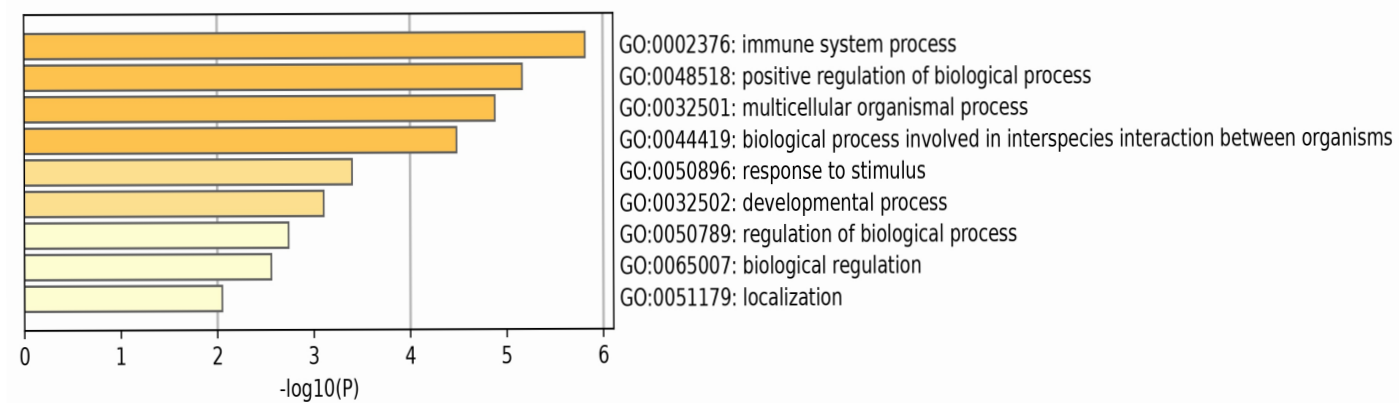

B

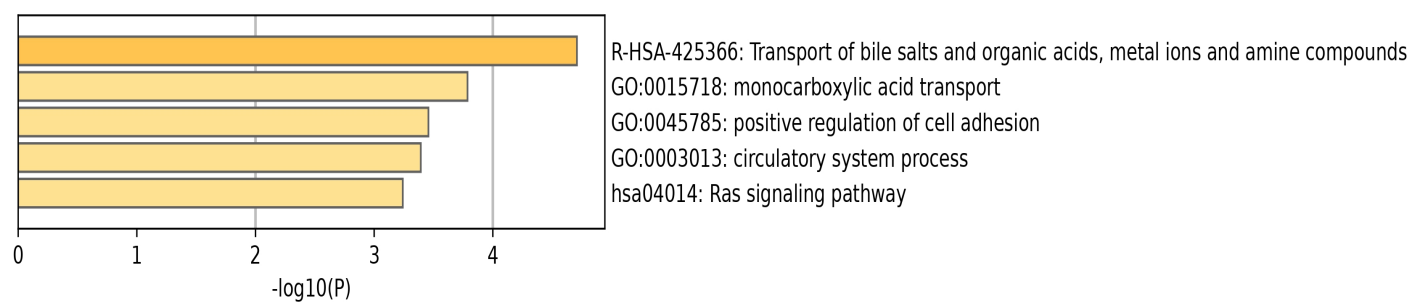

C

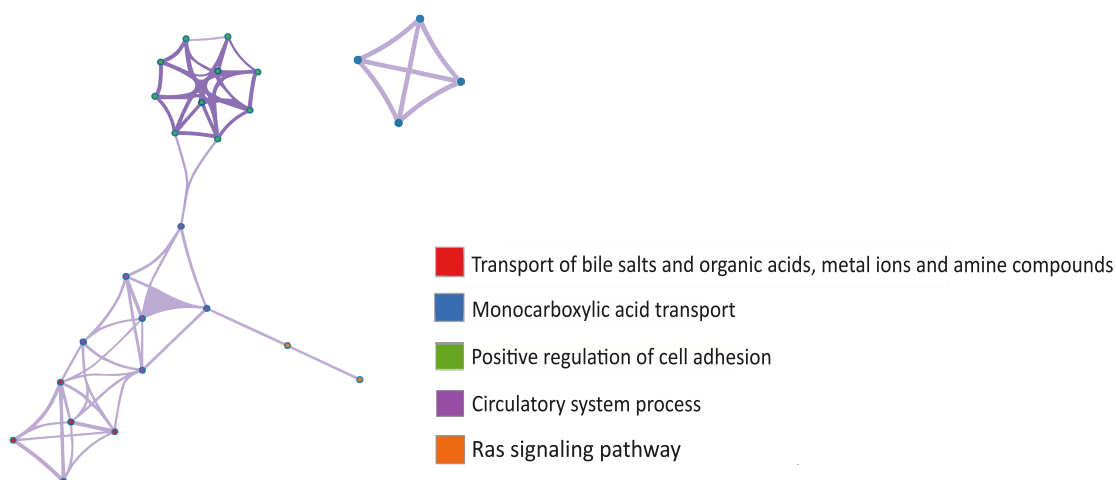

Supplement: Supplementary Figure 1 — Metascape pathway enrichment of common DEGs between YDC and ccRCC. [file DataSheet1.zip › Supplementary/Supply Figure 1.pdf]

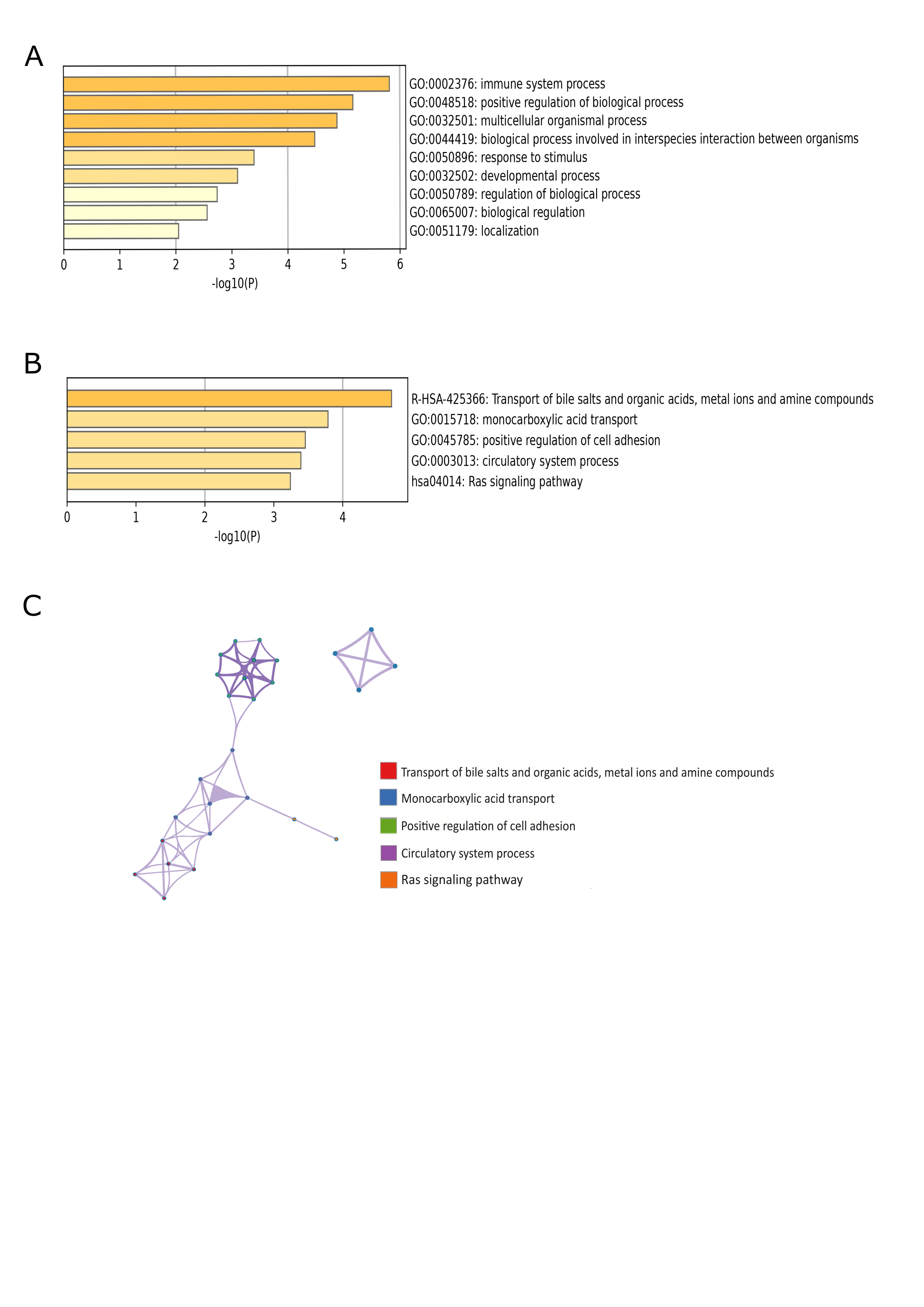

Supplement: Supplementary Figure 1 — Metascape pathway enrichment of common DEGs between YDC and ccRCC. [file DataSheet1.zip › Supplementary/Supply Figure 1.png]

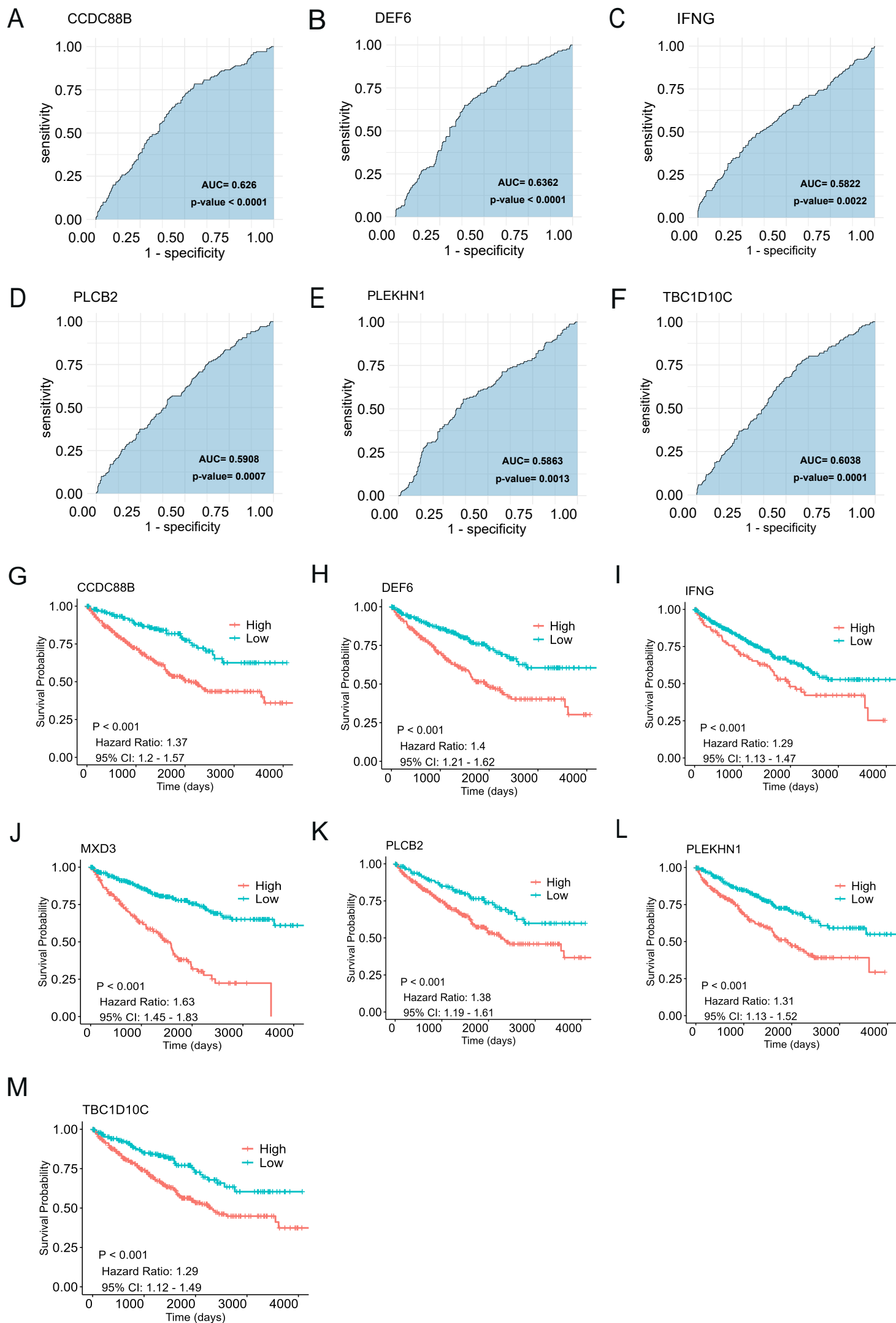

Supplement: Supplementary Figure 1 — Metascape pathway enrichment of common DEGs between YDC and ccRCC. [file DataSheet1.zip › Supplementary/Supply Figure 2.pdf]

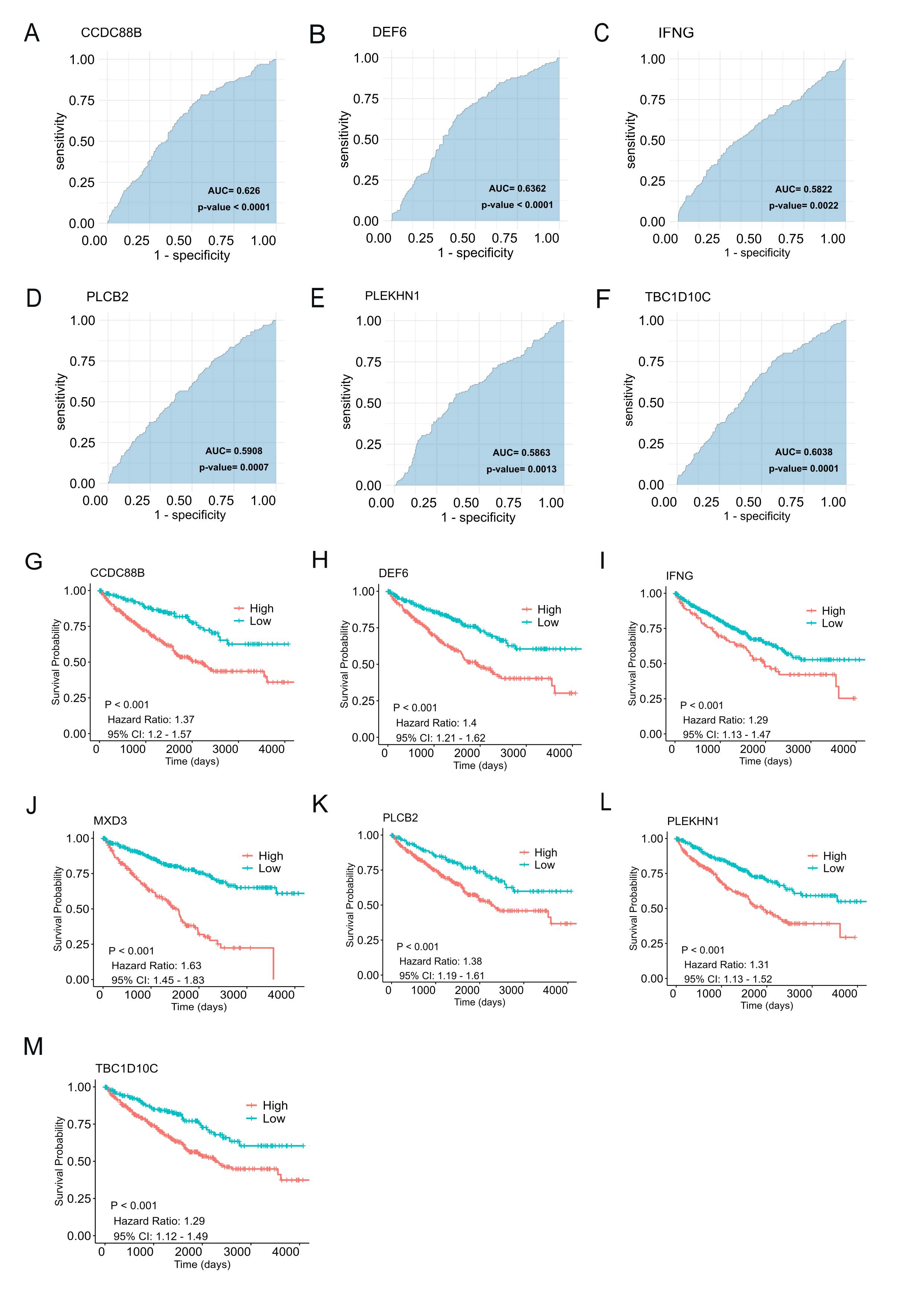

Supplement: Supplementary Figure 1 — Metascape pathway enrichment of common DEGs between YDC and ccRCC. [file DataSheet1.zip › Supplementary/Supply Figure 2.png]

**A**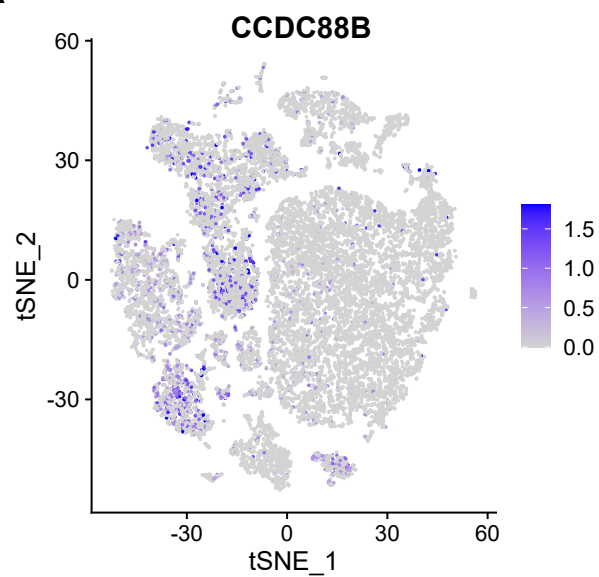**B**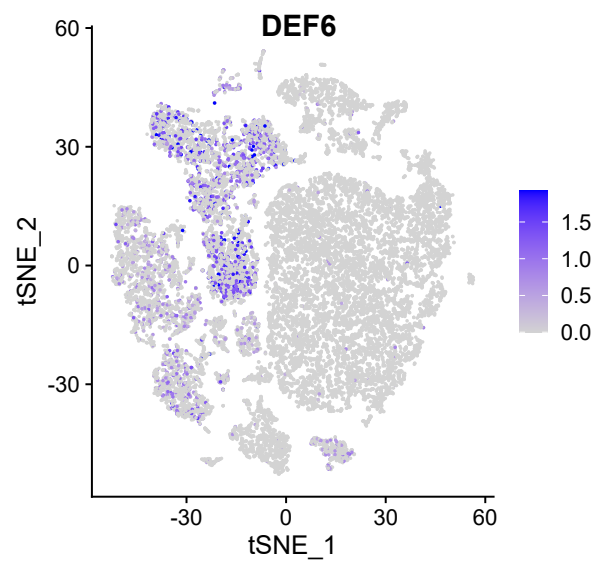**C**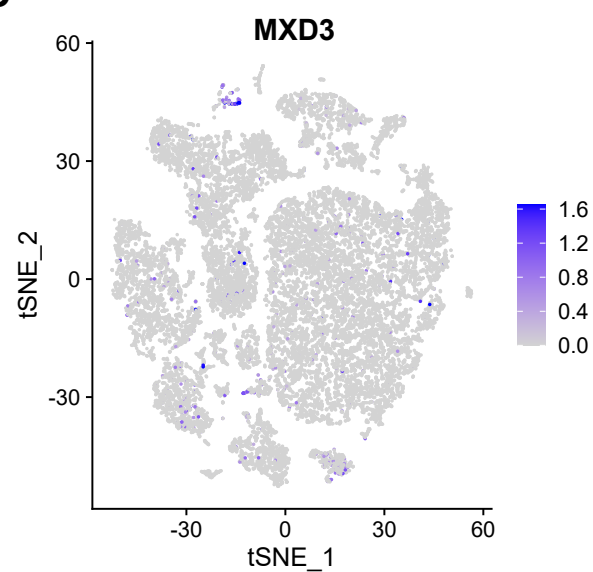**D**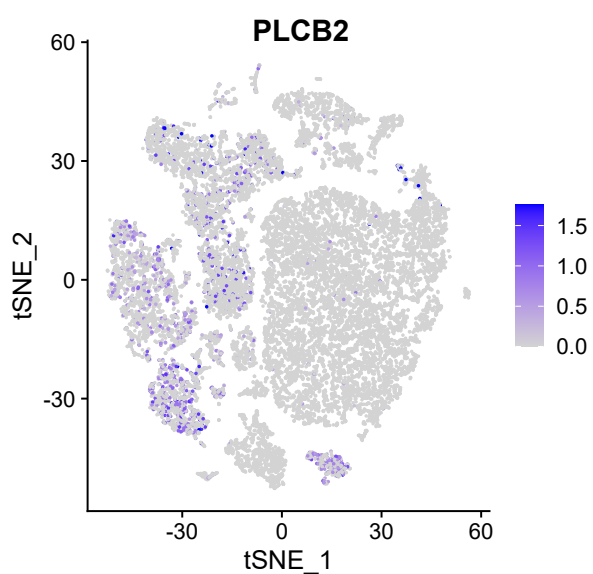**E**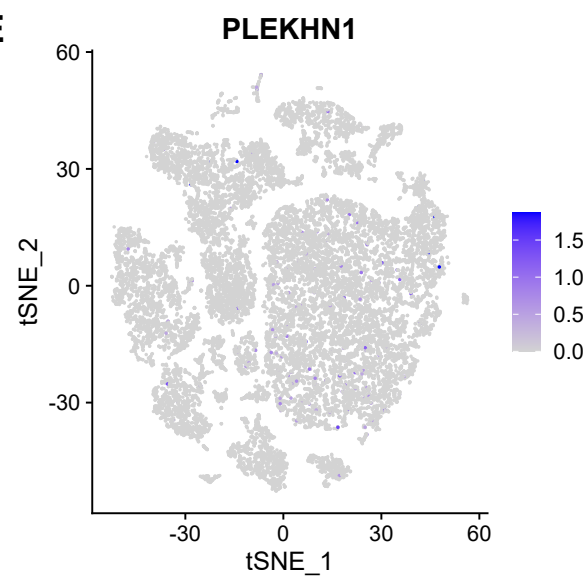**F**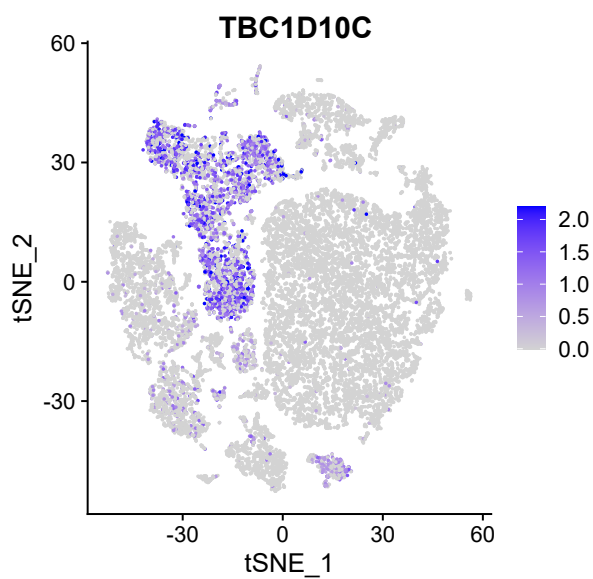

Supplement: Supplementary Figure 1 — Metascape pathway enrichment of common DEGs between YDC and ccRCC. [file DataSheet1.zip › Supplementary/Supply Figure 3.pdf]

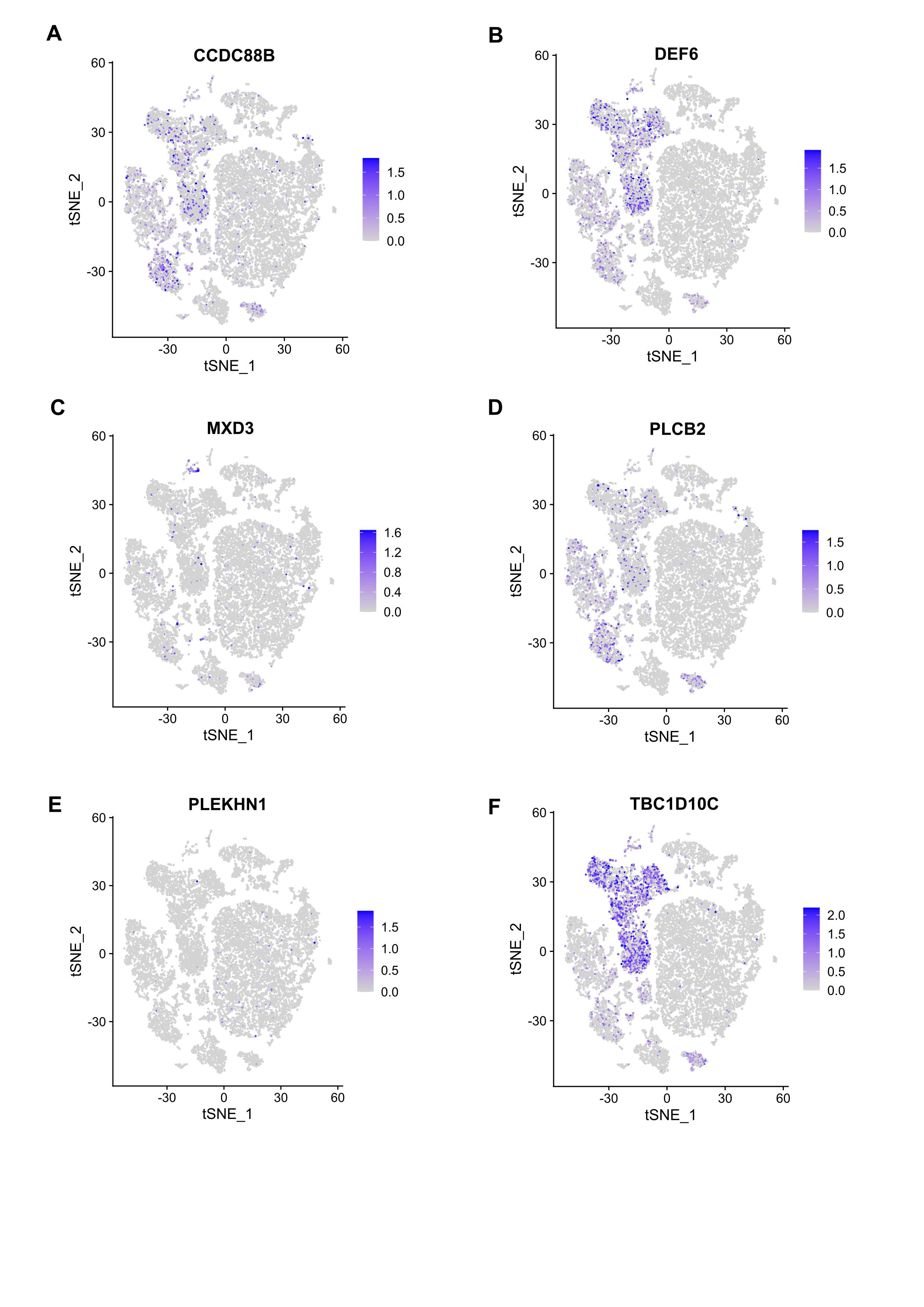

Supplement: Supplementary Figure 1 — Metascape pathway enrichment of common DEGs between YDC and ccRCC. [file DataSheet1.zip › Supplementary/Supply Figure 3.png]
